# A Brief Overview of Cholinergic and Phosphodiesterase-5 Inhibitors in Diabetic Bladder Dysfunction

**DOI:** 10.3390/ijms251910704

**Published:** 2024-10-04

**Authors:** Georgios Kallinikas, Georgios Haronis, Eirini Kallinika, Diomidis Kozyrakis, Evangelos Rodinos, Athanasios Filios, Panagiotis Filios, Despoina Mityliniou, Konstantinos Safioleas, Anastasios Zarkadas, Dimitrios Bozios, Athanasios Karmogiannis, Vasileios Konstantinopoulos, Anna Maria Konomi, Amin M. Ektesabi, James N. Tsoporis

**Affiliations:** 1 Department of Urology, Konstantopouleion–Patision Hospital, N. Ionia, 14233 Attika, Greece; georgioskallinikas@gmail.com (G.K.); george.xarwnhs@gmail.com (G.H.); dkozirakis@yahoo.gr (D.K.); vag.international@hotmail.com (E.R.); athanfilios@gmail.com (A.F.); panosfilios@yahoo.gr (P.F.); dmitiliniou@yahoo.gr (D.M.); konstantinossafioleas@yahoo.gr (K.S.); azark13@hotmail.com (A.Z.); dbozios@gmail.com (D.B.); krmgti@hotmail.com (A.K.); vkonstantinopoulos@yahoo.com (V.K.); annakonomi24@gmail.com (A.M.K.); 2 Department of Molecular Biology and Genetics, Democritus University of Thrace, 68100 Alexandroupolis, Greece; eirinikallinika@gmail.com; 3 Keenan Research Centre for Biomedical Science, Li Ka Shing Knowledge Institute, St. Michael’s Hospital, Unity Health Toronto, Toronto, ON M5B 1W8, Canada

**Keywords:** diabetic bladder, acetylcholine, muscarinic receptors, PDE5, anti-cholinergic drugs, PDE5 inhibitors

## Abstract

Diabetic bladder dysfunction (DBD) comprises a wide spectrum of lower urinary tract symptoms that impact diabetic patients’ lives, including urinary frequency, urgency, incontinence, and incomplete bladder emptying. To relieve symptoms, anticholinergics have been widely prescribed and are considered an effective treatment. There is increasing evidence that diabetic patients may benefit from the use of phosphodiesterase 5 (PDE5) inhibitors. This narrative review aims to provide a brief overview of the pathophysiology of DBD along with a focus on cholinergic and phosphodiesterase inhibitors as therapies that benefit DBD. An examination of the literature suggests compelling avenues of research and underscores critical gaps in understanding the mechanisms underlying DBD. New tools and models, especially rodent models, are required to further elucidate the mechanisms of action of current therapies in the treatment of DBS.

## 1. Introduction

According to the World Health Organization (WHO), 422 million people worldwide have diabetes mellitus (DM), and 1.5 million deaths per year are directly attributed to diabetes. The incidence of diabetes has steadily increased in recent decades and is expected to further increase. Diabetic bladder dysfunction (DBD) is probably the most common manifestation of the disease. DBD is thought to affect up to 60–80% of individuals with diabetes and can significantly impact patients’ quality of life [1,2,3]. These patients experience lower urinary tract symptoms (LUTSs) such as high frequency, decreased emptying or fullness sensation, increased post-micturition residual volume, urinary urgency, and incontinence [4,5]. Therefore, the early identification of risk factors for DBD and prediction of its development are crucial. Interestingly, HbA1c has been identified as a risk factor and potential biomarker for DBD [6].

DBD symptoms have been attributed to myogenic and neurogenic alterations. There are several theories on why this could be happening, such as hyperglycaemia-induced polyuria through hyperosmotic diuresis, the long-term accumulation of toxic metabolites, neuronal dysfunction, alteration of urothelium, and changes in bladder’s detrusor functionality; however, the underlying pathway(s) of DBD are not yet fully understood [4]. However, the symptoms mostly take place in patients with uncontrolled diabetes.

Voiding under physiological conditions is under the control of the parasympathetic nervous system. The best-studied neurotransmitter involved is acetylcholine. Acetylcholine binds to M2/M3 muscarinic receptors; this initiates the pathway signaling system that will cause the bladder’s smooth muscles (BSMs) to contract and the bladder to empty. Changes in BSM contractility are evident in the DBD of humans as well as animal models [7,8]. Data regarding detrusor contractile response to muscarinic agonists in diabetic animal models are inconclusive as increased [1], decreased [9], and unchanged activity has been reported [10]. The disruption of the insulin signaling pathway as it relates to DBD pathogenesis on a molecular level remains mostly unknown.

## 2. Materials and Methods

This literature review was conducted on PubMed, ResearchGate, and Google Scholar databases using the search terms “diabetic bladder”, “acetylcholine”, “muscarinic receptors,” and “PDE5” with no time restrictions. Initially, 184 publications were retrieved. Following a screening process, 135 articles were excluded for not meeting the selection criteria. An additional 7 articles were included based on references. Under the supervision of three authors (G.K., G.H., E.K.), the remaining articles underwent a full-text assessment. Finally, 66 articles were selected based on consensus under the management of two co-authors (G.K., J.N.T.).

## 3. Results

### 3.1. Muscarinic Receptors and Anti-Cholinergic Drugs in DBD

The neurotransmitter acetylcholine is involved in the activation of ionotropic nicotinic and metabotropic muscarinic receptors both in the periphery (peripheral?) and central nervous system [11]. In a systematic review, Caulfield et al. [12] showed that muscarinic receptors in the periphery mediate a plethora of actions including the contraction of smooth muscle, glandular secretion, and the modulation of heart rate. In the central nervous system, muscarinic receptors modify motor control, the regulation of temperature, and the fine-tuning of cardiovascular activity. Their diverse roles have spurred interest in muscarinic receptors, especially for potential therapies for diseases like Alzheimer’s, Parkinson’s, asthma, pain, and various cardiac and urinary disorders [12].

There are five distinct muscarinic receptor subtypes (M1–M5), though their exact locations and functions are not understood (Table 1). These receptors play vital roles within the same system, with signaling overlap. Caulfield et al. [12] reported that M1, M3, and M5 receptors couple to G (e.g., Gq/11, Ga, Gi) proteins, stimulating phospholipase C, releasing calcium from intracellular stores, and provoking extracellular calcium influx. Peralta et al. [13] showed that M2 and M4 receptors couple to Gi proteins, inhibiting adenylyl cyclase, reducing cAMP levels, and inducing smooth muscle relaxation. Other pathways may also be activated by M2 receptors.

**Figure 1:**
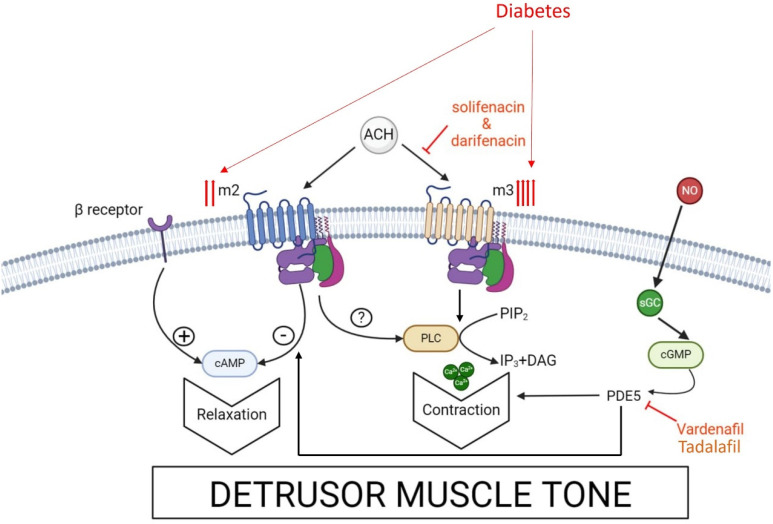
Schematic illustration of signaling pathways affecting detrusor muscle tone in diabetes and the effects of cholinergic and PDE5 inhibitor(s). Stimulation of β (3) receptors by norepinephrine activates AC with consequent increase in cAMP, which is degraded by PDEs to 5-AMP, and detrusor muscle contraction. Stimulation of muscarinic receptors reduces the cAMP level. M2/M3 receptor stimulation by ACH can activate PLC–calcium-dependent signaling, contributing to detrusor muscle contraction. NO generated by NO synthases activates sGC, increasing the intracellular concentration of cGMP. In turn, cGMP is degraded by PDEs leading to detrusor muscle contraction. Diabetes may increase muscarinic receptor density resulting in an amplification of the above signaling pathways resulting in pronounced detrusor muscle contraction. The targets of anti-cholinergic agents (moliferacin, dariferacin) and the PDE5 inhibitor (vardenafil) are shown. PDEs, phosphodiesterases; AC, adenylate cyclase; M, muscarinic receptor; AMP, adenosine monophosphate; PLC, phospholipase C; ACH, acetylcholine; NO, nitric oxide; GMP, guanosine monophosphate; sGC, soluble guanlyate cyclase; DAG, diacylglycerol; IP, inositol phosphate; PIP2, phosphatidylinositol 4,5-biphosphate.

Human detrusor contractility is under the control of the parasympathetic nervous system, with acetylcholine acting on muscarinic receptors. Wang et al. [19] studied tissues from rats, rabbits, guinea pigs, and humans, showing that all muscarinic receptor subtypes are present in detrusor muscle, with M2 and M3 being predominant (3:1). Hedge et al. [17] confirmed these findings. However, Fetscher et al. [14] found that M3 receptors primarily mediate human detrusor contraction in vitro using human tissue from cystectomy patients. There is evidence showing that the human bladder detrusor has a higher affinity for M3 receptors. Matsui et al. [15] found that in M3 knockout mice, 95% of carbachol-induced contraction is mediated by M3 receptors. The role of the abundant M2 receptors in detrusor muscle remains unclear. Ehlert et al. [16], using M2, M3, and double knockout mice, suggested that M2 receptors may enhance M3-mediated bladder contractions and may also mediate minor contractions directly. The contraction of the bladder’s detrusor is generated from the binding of acetylcholine on postjunctional membrane muscarinic M3 receptors, which then leads to the activation of the contractile proteins within the detrusor muscle [20]. Prejunctional M2 and M4 receptors inhibit, while the prejunctional M1 receptors facilitate acetylcholine release. The M2 receptors also appear to have an indirect functional role in detrusor contractility, but the exact mechanism of action remains unclear. Atropine inhibits contraction by blocking the muscarinic receptors (Figure 1) [18].

The pathogenesis of diabetes-induced bladder dysfunction, or diabetic cystopathy, is multifactorial, involving nerve dysfunction, urothelium alterations, and changes in detrusor muscle physiology [21]. The detrusor muscle is crucial for micturition, undergoing various changes such as modulation of the intercellular network, receptor density and distribution alterations, and genetic changes [21]. However, the evidence remains inadequate and contradictory, mostly based on animal studies. Muscarinic agonists enhance detrusor muscle sensitivity in diseases like DM [22], bladder outflow obstruction [23], and aging [24]. There is an increase in M2 receptor density in the diabetic rat bladder, suggesting a significant role in contractility, though few human studies exist [25]. Enhanced detrusor response to muscarinic agonists in diabetes may result from increased receptor density or smooth muscle sensitivity to calcium [26]. Tong et al. [27] studied three-month-old male Wistar rats, dividing them into two groups: 2-week-old diabetics and normoglycemic controls and showed a significant increase in M2protein (40.0 ± 6.2%, *p* < 0.05) and mRNA (69.3 ± 8.5%, *p* < 0.05) in diabetic bladders, indicating the upregulation of M2 biosynthesis within 2 weeks of diabetes induction. This increase may cause heightened acetylcholine reactivity and detrusor instability. Interestingly, Kubota et al. [28] found enhanced β1-receptor-mediated relaxation in detrusor muscle from rats 8–10 weeks post-diabetes induction with streptozotocin. Early diabetes augments muscarinic receptor density in rat bladders [27]. M3 muscarinic receptors cause bladder contraction via a PLC-independent mechanism [29], leading to IP3 activation [30], which induces Ca^2+^ release and muscle contraction [31]. Numerous studies have shown elevated intracellular calcium concentration and altered calcium channel sensitivity in diabetic rats [26].

All the anti-cholinergic drugs prescribed for DBD are taken by mouth and are absorbed in the small intestine, and metabolized in the liver through the P450 cytochrome system. Some of the most common side effects of this pharmacological category are dryness of mouth, constipation, dyspareunia, anhidrosis, blurred vision, urinary retention, arrythmias, etc., with elderly patients being more prone to experiencing side effects [32].

Belis et al. [33] supported these findings using nifedipine (a calcium channel antagonist) and BAY K8644 (an agonist) on the neurogenic responses of diabetic rats’ bladders. They examined the bladder body and base separately, finding no differences in the bladder bodies but an increased contractile response in the diabetic bladder bases compared to controls. Despite the absence of extracellular calcium, the diabetic bladder body and base showed increased contractility. Differences were observed in maximum responses between diabetic and control tissues treated with nifedipine and BAY K8644, with BAY K8644 failing to fully reverse nifedipine’s effects in diabetic bladders. These results suggest diabetes affects the activity of calcium channels in the bladder smooth muscle.

The effect of muscarinic receptor antagonists in patients with overactive bladder (OAB) has been extensively compared between those with and without concomitant diabetes. Scheider et al. [34] conducted an observational study with 532 diabetic OAB patients and 1315 non-diabetic OAB patients, all prescribed darifenacin. Diabetes was associated with less improvements in urgency, incontinence, frequency, and nocturia, but did not affect overall symptom improvement by darifenacin [34].

Höfner et al. [35] examined 741 men with OAB and LUTSs suggestive of non-obstructive benign prostatic hyperplasia (BPH). Patients were treated with 4mg/day tolterodine extended-release alone or added to an existing partially successful α-blocker treatment. Among various subgroups, mean total international prostate symptom scores (IPSSs) improved by 2.8–11.1 points, IPSS quality of life (QOL) scores by 1.8–2.4 points, and all OAB sub-scores by more than 14 points. The severity of baseline symptoms was the main predictor of treatment benefit, with more severe symptoms showing greater improvement. The addition of tolterodine provided comparable improvements in IPSSs for both diabetic and non-diabetic men [35].

Obata et al. [36] investigated solifenacin add-on therapy to tamsulosin in 130 BPH patients with persistent OAB symptoms. After 8 weeks of solifenacin 5 mg/day, improvements in IPSS, QOL index, and overactive bladder symptom score (OABSS) were observed. Prostate volume was the main predictor of IPSS improvement, with patients having <30 mL showing better results. Diabetes was a considerable factor preventing OABSS improvement, with diabetic patients showing less improvement compared to non-diabetics [36].

Choi et al. [37] conducted a 12-week study comparing solifenacin’s effects in 81 women with diabetes-associated OAB, and 160 with idiopathic OAB with no baseline differences, and assessed alterations in OABSS, urgency, incontinence, nocturia, and daytime frequency. Solifenacin treatment improved urinary urgency incontinence, nocturia, frequency (per voiding diary), and total OABSS between visits V1 and V2, and V1 and V3. In the diabetes OAB group, improvements in urgency and urge incontinence were noted between V2 and V3 [37]. Despite small sample sizes complicating interpretation, it appears that diabetes reduces the efficacy of muscarinic antagonists in OAB patients. However, these antagonists may still show some clinical effects in diabetic patients with OAB. Importantly, this study showed that even in the absence of BPH, solifenacin improved OAB symptoms.

Oger-Roussel et al. [38] conducted an animal study on type 2 diabetic Goto-Kakizaki (GK) rats and age-matched Wistar rats. They assessed bladder function with cystometry and erectile function after administering solifenacin and sildenafil. GK rats showed detrusor overactivity, with increased frequency/amplitude of non-voiding contractions, bladder capacity, inter-contraction interval, voided volume, and maximal voiding pressure. Solifenacin reduced non-voiding contractions without affecting voiding efficiency. Despite using sildenafil, GK rats showed no improvement in erectile function, making GK rats a suitable model for studying diabetes-related bladder and erectile dysfunction [38].

Lua et al. [39] evaluated adherence and persistence of anticholinergic medications in OAB, focusing on gender and obesity, using the Truven Marketscan database (2005–2013). The study enrolled 122,641 OAB patients aged 18–65 for ≥12 months. Adherence was measured by medication possession ratio and proportion of days covered, while persistence was defined as the duration from initiation to discontinuation, switch, or study exit. Hypertension, depression, and diabetes were common comorbidities, with affected patients showing higher compliance. Obese patients were 7% less likely to adhere and 6% more likely to become non-compliant compared to non-obese patients. Men were about 20% more likely to adhere to anticholinergics than women. Tolterodine, oxybutynin, and solifenacin were the most common anticholinergics, with solifenacin showing the highest adherence and compliance. After one year, 17.11% of patients remained on solifenacin compared to 12.64% for all anticholinergics combined. The study results suggest that the slightly lower efficacy of OAB medication in diabetic patients might be due to adherence issues [39]. The lower efficacy of muscarinic antagonists in obese patients, who often have diabetes, may be due to lower compliance rather than pharmacodynamic differences [39].

Selig et al. [40] conducted a retrospective cohort study on females aged 18 and older within the Military Health System from 2006 to 2016, examining the link between antimuscarinic medication and type 2 (T2) DM risk. Patients without prior T2DM history using anticholinergic drugs (tolterodine, oxybutynin, fesoterodine, solifenacin, darifenacin, trospium) were followed for T2DM occurrence. The results showed that patients exposed to M3 selective antagonists (solifenacin and darifenacin) had a higher risk of developing T2DM compared to those using nonselective ones (oxybutynin). However, it remains unclear whether darifenacin or solifenacin cause or are less effective in preventing diabetes compared to oxybutynin.

### 3.2. PDE5 Activity and PDE5 Inhibitors in DBS

During sexual arousal, nitric oxide is released from nerve terminals and the endothelium of the corpus cavernosum, initiating a signaling cascade that activates guanylate cyclase, converting guanosine triphosphate into cyclic guanosine monophosphate (c-GMP). Increased c-GMP levels lead to smooth muscle relaxation. Phosphodiesterase type 5 (PDE5) degrades c-GMP to inactive 5′-cGMP, inhibiting muscle relaxation [39] (Figure 1). PDE5 inhibitors, structurally similar to c-GMP, bind to PDE5 and prevent c-GMP degradation [41].

Immunohistochemical studies show PDE5 presence in male lower urinary tract muscle fibers but not in the epithelium, with higher PDE5 mRNA expression in the bladder than in prostate smooth muscle cells [42]. PDE5 is one of eleven PDE families that hydrolyze cyclic adenosine monophosphate and c-GMP, playing important roles in various physiological and pathological pathways by controlling their concentrations. Different PDE subtypes are found in animal and human bladders [43]. In rats, PDE5 expression and activity in the bladder are regulated by testosterone [44]. PDE5 is also present in the urothelium, suburothelia interstitial cells, and bladder blood vessels of guinea pigs [45].

PDE5 inhibitors, which have been certified for the treatment of erectile dysfunction, pulmonary hypertension, LUTSs associated with BPH, and penile rehabilitation after radical prostatectomy, are all taken by mouth, absorbed in the small intestine, and metabolized in the liver via CYP3A pathway [46]. All PDE5 inhibitors undergo extensive tubular reabsorption in the kidney and excreted as metabolites in the stool [46]. The most common side effects are headache, flushing, hypotension, and rhinitis. Co-administration with nitrates is contra-indicated. In several pre-clinical studies for off-label indications, PDE5 inhibitors show a beneficial impact on various systems including the musculoskeletal, neurological, cutaneous, and gastrointestinal systems [47].

The PDE5 inhibitor Vardenafil primarily acts on tissues in the bladder, rather than bladder nerves [48]. In the bladder of rabbits and humans, PDEs 1–5 are mainly found in detrusor smooth muscle, especially in the dome and lateral walls [48,49]. RT-PCR studies revealed mRNAs for PDEs 7–9 and 15, with PDE1 and PDE9 expressed in the urothelium. PDE5s are more concentrated in the bladder than in the urethra and prostate, with the highest expression in the lower urinary tract’s muscular cells [42]. PDE5s act on cGMP, affecting intracellular calcium concentration [50].

Nitric oxide is a crucial non-adrenergic, noncholinergic neurotransmitter that works with adrenergic and cholinergic systems to regulate micturition in the lower urinary tract [51]. Nitric oxide inhibits efferent neurotransmission in the urethra and regulates afferent neurotransmission in the bladder, along with related reflex pathways to and from the spinal cord [52,53]. When nitric oxide is released from nerve endings and the endothelium, it diffuses into smooth muscle cells, stimulating cGMP synthesis, leading to muscle relaxation. cGMP is then hydrolyzed to inactive 5′-GMP by PDEs [54]. Both cAMP and cGMP are involved in micturition control, playing roles in the pathophysiology of storage and voiding disorders [54,55,56].

PDE5 inhibitors are recommended for treating erectile dysfunction and have been evaluated for LUTSs (Table 2). Tadalafil has been approved for treating male LUTSs related to benign prostatic enlargement [57]. In a study by Matsuo et al. [58], male patients with urinary symptoms were prescribed tadalafil, meeting the criteria for OAB. DM was present in 10 of 44 responders but in none of the 9 non-responders, indicating tadalafil’s positive effects on LUTSs in DM patients [58].

Gotoh et al. [59] conducted an animal study using female Sprague Dawley rats, divided into non-diabetics, diabetics, and diabetics treated with tadalafil (2 mg/kg/day) for 7 days. Seven weeks post-induction, tadalafil restored prolonged inter-contraction intervals and bladder blood flow to control levels [59]. A follow-up study [60] confirmed these findings, showing tadalafil restored detrusor muscle contractility in diabetic rats. Masuda et al. [61] also demonstrated tadalafil’s restorative effects on detrusor function in diabetic Wistar rats.

Thurmond et al. [62,63] performed a systematic review indicating that tadalafil’s benefits might stem from improved bladder perfusion, reversing diminished perfusion in bladder dysfunction. Rybalkin et al. [50] reported that vardenafil, another PDE5 inhibitor, decreased bladder smooth muscle contractility by altering cGMP metabolism, and reducing intracellular calcium levels. Jong Soo Han et al. [64] found that the cGMP pathway is involved in bladder smooth muscle relaxation control in diabetes, with PDE5 inhibitors improving smooth muscle contractility.

## 4. Discussion

DM is a metabolic disease that affects millions of people worldwide. The number of people diagnosed with diabetes has risen steeply from 108 million in 1980 to 422 million today. Diabetes is a major cause of co-morbidities and impacts all systems of the human body. In 2019, the estimated number of deaths linked to diabetes was 2 million. Diabetic bladder dysfunction refers to a wide spectrum of bladder symptoms in patients with diabetes; it is one of the most common complications of diabetes that influences the urinary system and has a detrimental impact on patients’ quality of life [3,4]. Symptoms include decreased bladder sensation, increased bladder capacity, reduced bladder contractility, frequent urination, nocturia, urgency, and occasional dribbling, while the risk of infections is increased. It appears that the symptoms are related to the diabetic neuropathy; however, the molecular pathways are largely unknown [3,4].

Of the five different types of muscarinic receptors, only M2 and M3 appear to play an important functional role in the bladder [13,19]. The detrusor muscle of the bladder plays a major role in micturition and is also considered to be linked directly to many of the diabetic bladder symptoms. In diabetic patients, muscarinic agonists promote the functionality of the detrusor muscle [21,65]. This could be either attributed to an increased density of muscarinic receptors or an elevated sensitivity of the smooth muscle’s response to calcium [25] (Figure 1). Animal experiments have shown an increase in M2 receptors’ density and biosynthesis [26] in diabetes. These findings are in accordance with the outcomes of several studies that confirm that diabetic patients who experience DBS and are widely prescribed anticholinergics, while PDE5 inhibitors appear to be a promising alternative [33,34].

PDE5 is one of the eleven in the family of PDEs that hydrolyses cAMP and cGMP. PDEs have been found in the bladder of animals as well as humans. In humans, the PDEs are expressed in the detrusor smooth muscle, in the dome and lateral walls [45,66]. Available data from PDE5 inhibitors studies suggest a functional role of PDE5 in DBS (Figure 1). Gain of function and loss of function studies with transgenic and knockout mice, respectively, are warranted to unravel important PDE5 signal pathways [67].

In a study by Matsuo et al. [58], nearly 25% of male patients with diabetes who were treated with a PDE5 inhibitors for LUTSs found no benefit, and the respective percentage in non-diabetic patients was 0%. In an animal study by Gotoh et al. [59], the use of PDE5 inhibitors restored the impaired contractility of the detrusor muscle of the bladder in female diabetic rats. Although there appears to be a benefit in diabetic patients with DBD when using PD5 inhibitors, the exact signaling pathway(s) remain largely unknown. One hypothesis is that muscle cells of the bladder and vascular smooth muscle cells respond similarly to PDE. In particular, VSMC contractility is under the regulation of the cGMP-PDE5 pathway, the malfunction of which leads to vascular elasticity disorder and hypertension [68,69]. Interestingly, sildenafil increases the number of circulating endothelial progenitor cells (EPCs), the relative expression of C-X-C chemokine receptor 4 on these cells, and the ability to generate colonies in vitro, suggesting an involvement of PDE5 in bone marrow release, peripheral homing of EPC, and vascular repair [70]. Furthermore, it has been shown that the use of PDE5 inhibitors prevents the production of chemokine modulators [71,72].

## 5. Conclusions

In DBD, there appears to be an upregulation in both the density as well as sensitivity of M receptors, mostly M2 and M3, and DBD patients benefit from the use of anti-cholinergic agents. Additionally, some diabetic patients with DBD seem to benefit when prescribed PDE5 inhibitors through a largely unknown mechanism. The development of PDE5 rodent animal models (e.g., PDE5 transgenic/knockout mice) may shed some light on the signaling pathways involved.

## Figures and Tables

**Table 1:** Muscarinic receptor functions in the bladder.

Muscarinic Receptor	Method/Models	Results	Authors
M1 + M3 + M5	Systematic review	System of inositol trisphosphate (IP3) activation.	[12]
M2 + M4	Systematic Review	Reduction in cAMP levels—smooth muscle relaxation.	[13]
M3	Human tissue specimens	Affinity in human bladder detrusor is greater for the M3 subtype.	[14]
	M3 knockout mice	Contraction induced by carbachol mediated by M3 receptors.	[15]
M2 + M3	M2, M3, and M2/M3 double knockout mice	M2 receptors enhance contractile response to M3 receptors’ activation/minor M2 receptors’ impact on contractions.	[16]
M2	Review	M2 receptors inhibit smooth muscle relaxation.	[17]
M1 + M2 + M3 + M4 + M5	Systematic review	Binding of Acetylcholine on muscarinic M3 receptors (M3)/prejunctional M2 and M4 receptors inhibit the release of ach/prejunctional M1 receptors facilitate the release of Ach.	[18]

**Table 2:** PDE5 inhibitors and impact on diabetic bladder syndrome.

Drug	Models/Method	Results	Authors
Tadalafil	Human male participants	Symptoms improved in 10/44 DM patients.	[58]
Tadalafil	Female Sprague Dawley rats	Improvement in inter-contraction intervals and bladder blood flow.	[59]
Tadalafil	Female Sprague Dawley rats	Restored contractility of detrusor muscle.	[60]
Tadalafil	Male Wistar rats	Restored detrusor function.	[61]
Tadalafil	Systematic review	Improved perfusion of the bladder.	[62,63]
Vardenafil	Review article	Decreased contractility of bladder’s smooth muscle.	[50]
Udenafil	Male Sprague Dawley (SD) rats	Significant improvement in smooth muscle cell contractility.	[64]

## Data Availability

All data are presented in the manuscript.

## Abbreviations

BPH	benign prostate hyperplasia
BSM	bladder smooth muscle
c-GMP	cyclic-guanosine monophosphate
DBD	diabetic bladder dysfunction
DBS	diabetic bladder syndrome
DM	diabetes mellitus
GK	Goto-Kakizaki
IPSS	international prostate symptom score
LUTSs	lower urinary tract symptoms
OAB	overactive bladder
OABSS	overactive bladder symptom score
QOL	quality of life
PDE	phosphodiesterase
